# Olaparib and Ceralasertib (AZD6738) in Patients with Triple-Negative Advanced Breast Cancer: Results from Cohort E of the plasmaMATCH Trial (CRUK/15/010)

**DOI:** 10.1158/1078-0432.CCR-23-1696

**Published:** 2023-09-29

**Authors:** Alistair Ring, Lucy S. Kilburn, Alex Pearson, Laura Moretti, Angelica Afshari-Mehr, Andrew M. Wardley, Bora Gurel, Iain R. Macpherson, Ruth Riisnaes, Richard D. Baird, Sue Martin, Rebecca Roylance, Hannah Johnson, Ana Ferreira, Matthew C. Winter, Kathryn Dunne, Ellen Copson, Tamas Hickish, Russell Burcombe, Kat Randle, Violeta Serra, Alba Llop-Guevara, Judith M. Bliss, Nicolas C. Turner

**Affiliations:** 1 Breast Unit, The Royal Marsden Hospital, Sutton, United Kingdom.; 2 Division of Breast Cancer Research, Institute of Cancer Research, London, United Kingdom.; 3 Clinical Trials and Statistics Unit at The Institute of Cancer Research, London, United Kingdom.; 4 The Breast Cancer Now Toby Robins Research Centre, The Institute of Cancer Research, London, United Kingdom.; 5 Outreach Research & Innovation Group, Manchester, United Kingdom.; 6 Clinical Studies – Cancer Biomarkers, The Institute of Cancer Research, London, United Kingdom.; 7 School of Cancer Sciences, University of Glasgow, Glasgow, United Kingdom.; 8 Cancer Research UK Cambridge Centre, Cambridge, United Kingdom.; 9 University College London Hospitals NHS Foundation Trust & NIHR University College London Hospitals Biomedical Research Centre, London, United Kingdom.; 10 Weston Park Hospital, Sheffield Teaching Hospitals NHS Foundation Trust, Sheffield, United Kingdom.; 11 Ralph Lauren Centre for Breast Cancer Research, Royal Marsden Hospital, London, United Kingdom.; 12 Cancer Sciences Academic Unit, University of Southampton, Southampton, United Kingdom.; 13 Royal Bournemouth Hospital, University Hospitals Dorset NHS Foundation Trust, Bournemouth, United Kingdom.; 14 Maidstone and Tunbridge Wells NHS Trust, Maidstone, Kent, United Kingdom.; 15 Independent Cancer Patients’ Voice, London, United Kingdom.; 16 Experimental Therapeutics Group, Vall d'Hebron Institute of Oncology, Barcelona, Spain.

## Abstract

**Purpose:**

Approximately 10% to 15% of triple-negative breast cancers (TNBC) have deleterious mutations in *BRCA1* and *BRCA2* and may benefit from PARP inhibitor treatment. PARP inhibitors may also increase exogenous replication stress and thereby increase sensitivity to inhibitors of ataxia telangiectasia and Rad3-related (ATR) protein. This phase II study examined the activity of the combination of PARP inhibitor, olaparib, and ATR inhibitor, ceralasertib (AZD6738), in patients with advanced TNBC.

**Patients and Methods:**

Patients with TNBC on most recent biopsy who had received 1 or 2 lines of chemotherapy for advanced disease or had relapsed within 12 months of (neo)adjuvant chemotherapy were eligible. Treatment was olaparib 300 mg twice a day continuously and celarasertib 160 mg on days 1–7 on a 28-day cycle until disease progression. The primary endpoint was confirmed objective response rate (ORR). Tissue and plasma biomarker analyses were preplanned to identify predictors of response.

**Results:**

70 evaluable patients were enrolled. Germline *BRCA1/2* mutations were present in 10 (14%) patients and 3 (4%) patients had somatic *BRCA* mutations. The confirmed ORR was 12/70; 17.1% (95% confidence interval, 10.4–25.5). Responses were observed in patients without germline or somatic *BRCA1/2* mutations, including patients with mutations in other homologous recombination repair genes and tumors with functional homologous recombination deficiency by RAD51 foci.

**Conclusions:**

The response rate to olaparib and ceralasertib did not meet prespecified criteria for activity in the overall evaluable population, but responses were observed in patients who would not be expected to respond to olaparib monotherapy.

**Translational Relevance**

The standard systemic therapy of advanced Triple Negative Breast Cancer is cytotoxic chemotherapy, antibody-drug conjugates and immunotherapy (in those with PDL-1 positive tumor). Despite more recent developments, the median overall survival of such patients is 2 years. PARP inhibitors are indicated in the 10–15% of patients with germline or somatic BRCA mutations. Cohort E of the plasmaMATCH Trial found that when a PARP inhibitor (Olaparib) was combined with an ATR inhibitor (Celerasertib), responses were seen both in patients with BRCA mutations and in those without. Patients with pathogenic mutations in other homologous recombination repair genes and low RAD51 foci were amongst responders. This means that patients without BRCA mutations, identifiable by other molecular predictors, may be able to benefit from this therapeutic approach.

## Introduction

Approximately 10% to 15% of breast cancers do not express hormone receptors [estrogen receptor (ER) or progesterone receptor (PgR)] and are HER2-negative. This triple-negative breast cancer (TNBC) subgroup has a poor prognosis with overall survival for those patients with metastatic disease of 1 to 2 years (1). The identification of novel therapeutic targets and treatment approaches in patients with this breast cancer subtype is therefore paramount. The PARP enzyme is required for single strand break DNA repair, and cancer cell lines with defective homologous recombination (HR) are unable to tolerate the DNA damage that results from PARP inhibition, resulting in cell-cycle arrest and apoptosis. Both *BRCA1* and *BRCA2* are needed for HR and consequently cancer cell lines deficient in *BRCA1* or *BRCA2* are highly sensitive to PARP inhibitors. Approximately 10% to 15% of TNBCs have deleterious mutations in *BRCA1* and *BRCA2*, and the PARP inhibitors olaparib and talazoparib are licensed for the treatment of advanced HER2-negative breast cancer associated with these mutations. In addition, a proportion of sporadic TNBCs may have defective HR, in part due to reduced *BRCA1* expression or *BRCA1* promoter methylation, that may therefore benefit from PARP inhibitor treatment (2–4).

AZD6738 (cerelasertib) is a potent, selective inhibitor of the serine/threonine-specific protein kinase, ataxia telangiectasia and Rad3-related (ATR) protein. ATR is an apical kinase in one of the DNA-damage induced checkpoint pathways (5). During normal DNA replication ATR is recruited at stalled replication forks, which can progress to double-strand breaks (DSB) if left unrepaired. Recruitment and activation of ATR leads to cell-cycle arrest in the S-phase while the DNA is repaired; either the stalled replication fork is resolved, or there is nuclear fragmentation and apoptosis. Loss of ATR function leads to the inability to resolve stalled replication forks, resulting in the accumulation of DNA damage and cell death. Increasing the exogenous replication stress in combination with PARP inhibitors such as olaparib could increase the sensitivity of ATR inhibitors. The combination of olaparib with an ATR inhibitor is therefore hypothesised to be active in TNBC (6). Ataxia telangiectasia–mutated (ATM serine/threonine kinase) is a further apical kinase of DNA DSB response, that signals in a partially nonredundant fashion with ATR, and ATR inhibitors have synthetic lethal activity in ATM-deficient cancers (7). Preclinical models have shown that TNBC, *BRCA* mutant and ATM-deficient cancer may be highly sensitive to the combination of PARP inhibitors and DNA damage response kinase inhibitors such as ATR inhibitors (8–10).

The plasmaMATCH trial was an open label, non-randomized, phase IIa clinical trial platform, consisting of circulating tumor DNA (ctDNA) testing in patients with advanced breast cancer linked to parallel treatment cohorts with therapies matched to mutations identified in ctDNA. The ctDNA screening component of the trial and Cohorts A–D have already been reported (11). With the low incidence of the mutations targeted (*ESR1, ERBB2, AKT1, PTEN*) in TNBC, an additional cohort, Cohort E, was added later to the adaptive trial platform to include patients with TNBC without a targetable mutation identified at ctDNA screening, treating them with olaparib (PARP inhibitor) plus ceralasertib (ATR inhibitor, formerly AZD6738). Here we report the principal results of Cohort E, along with predefined biomarker subgroup analyses.

## Patients and Methods

### Patients

Cohort E of the plasmaMATCH study was designed to recruit patients with advanced TNBC as determined on their most recent biopsy. TNBC defined as ER-negative, PgR-negative (ER- and PgR-negative defined as either Allred score 0/8 or 2/8 or stain in <1% of cancer cells) or ER-negative, PgR unavailable, and HER2-negative (IHC 0/1+ or negative in situ hybridization) as determined by local laboratory. These patients had undergone ctDNA screening as part of plasmaMATCH trial registration and were not able to enter Cohorts A–D because either no actionable mutations were identified; or an actionable mutation was identified but the cohort was closed; or the patient did not meet the relevant cohort specific eligibility criteria. Eligible patients had an Eastern Cooperative Oncology Group performance status of 0 or 1 and were suitable for a baseline advanced disease biopsy or had an archival advanced disease biopsy available for translational analyses. Patients had disease progression by radiological assessment and had completed at least one prior line of treatment for advanced breast cancer and/or relapsed within 12 months of (neo)adjuvant chemotherapy. A maximum of two prior lines of chemotherapy, antibody–drug conjugate or immunotherapy for advanced disease were permitted. If patients had received a prior platinum-containing therapy for metastatic disease they were required to have achieved a partial response (PR)/complete response (CR) or stable disease (SD) and not have progressed during or within 8 weeks of receipt of last dose of platinum.

### Treatment and procedures

Details on ctDNA testing have been reported previously (11). Treatment for patients eligible for Cohort E was with olaparib 300 mg twice daily administered orally on each day of the treatment cycle and ceralasertib 160 mg once daily administered orally on days 1–7 of each 28-day treatment cycle. Treatment was until disease progression or unacceptable toxicity. Participants could also discontinue from trial treatment at any time at their own request or be discontinued at the discretion of the treating clinician. Dose modifications were permitted for patients experiencing toxicities related to treatment. Patients underwent CT or MRI scan and bone scan at baseline, with CT or MRI scan repeated 8 weekly until 32 weeks and 12 weekly thereafter. Laboratory assessments, adverse event (AE) recording and vital signs were performed at least every 4 weeks. Toxicity was assessed using NCI Common Terminology Criteria for Adverse Events version 4. Coding was done with use of the Medical Dictionary for Regulatory Activities (MedDRA) version 22.

### Endpoints

The primary endpoint was confirmed objective response rate (ORR) defined as a confirmed CR or PR at any point during trial treatment according to RECIST criteria, version 1.1. Secondary endpoints included duration of response (defined as time from the first documentation of CR or PR until date of disease progression or last date of follow-up), clinical benefit rate (defined as CR, PR, or SD for more than 6 months during trial treatment), progression-free survival (PFS; defined as time from cohort entry to first date of either confirmed progression of disease according to RECIST criteria or death from any cause), safety and tolerability of therapies.

### Statistical considerations

A Simon two-stage design was used with a target initial response rate of 25% and unacceptable response rate of 10%, two-sided α = 0.02 and 90% power. Stage 1 required recruitment of 37 evaluable patients and at least 5 responses to be observed to continue recruitment to a total of 69 evaluable patients where at least 13 responses were required to infer sufficient activity of the olaparib–ceralasertib combination.

ORR, duration of response, and clinical benefit rate were measured in an evaluable population defined as those patients with measurable disease per RECIST at baseline and at least one on-treatment assessment; patients who stopped treatment because of intolerable toxicity or death without having a scan after baseline were evaluable and recorded as nonresponders. Proportions and two-sided 95% confidence intervals (CI) for estimation purposes were reported. For the primary endpoint, in addition to the response rate reported as a percentage (responses/number of evaluable patients), the uniformly minimum-variance unbiased estimator (UMVUE) and adjusted 95% CI are also reported to account for the two-stage design (12, 13). Subgroup analyses were planned to analyze activity according to *BRCA* status (germline and somatic) and homologous recombination repair (HRR) genes (from ctDNA). Analyses by *BRCA1/2* mutation status; and, in patients with no germline or somatic *BRCA1/2* mutations, by ATM loss (“loss” defined as Hscore ≤10), Cyclin E1 (high/low; cutpoint = median) and RAD51 foci formation (high/low; low ≤10%) were conducted. No formal comparisons between subgroups were made.

PFS used the intention-to-treat population. Kaplan–Meier curves were plotted and median PFS was reported with 95% CI. Patients who were alive and progression-free were censored at date of last follow-up; patients who had non-RECIST confirmed progression (e.g., clinical progression only or radiologically confirmed but lesions not measured according to RECIST) were censored at the date progression was reported. The safety population included all patients who had at least one dose of treatment and treatment-emergent AEs where >10% patients reported any grade or any patients reporting grade ≥3 were presented.

Analyses used a database snapshot taken on June 15, 2021. Where reported, *P* values of less than 0.05 were deemed significant. All analyses were conducted using Stata (version 16) and R (version 4.1.1).

### Translational analyses

All patients provided a new or archival tissue biopsy from recurrent disease. Blood samples were taken for germline analysis (baseline only) and for biomarker/ctDNA analysis (baseline and pretreatment cycles). *BRCA1/2* germline mutation analysis was performed by local testing, or central lab developed test if no local result were available. *BRCA1/2* somatic mutation analysis was performed using baseline Guardant360, and HRR gene analysis (*ARID1A, ARID2, ATRX, BAP1, CDK12, CHEK1, CHEK2, FANCD2, NBN, RAD51D, SMARCA4, FANCM PALB2*) with baseline Guardant OMNI (Guardant Health; Redwood City, CA). Only pathogenic/likely pathogenic mutations were reported, filtered as previously reported (14). ATM IHC analysis was with clone Y170, negative with H score ≤10 (15). Cyclin E1 IHC analysis was with clone HE12, percentage of positive nuclei scored, split positive-negative by median (16). RAD51 immunofluorescence was with ab133534, assessed in geminin positive cells 10802–1-AP, and low RAD51 (≤10%) identified HR-deficient tumors (17).

### Study oversight

The study was cosponsored by The Institute of Cancer Research and the Royal Marsden NHS Foundation Trust and approved by a Research Ethics Committee (16/SC/0271). The study was conducted according to the approved protocol and its amendments, supplementary guidance and manuals supplied by the cosponsors and in accordance with The Medicines for Human Use (Clinical Trials) Regulations 2004 as amended, the Research Governance Framework for Health and Social Care and the principles of Good Clinical Practice. All participants gave written informed consent prior to registration for ctDNA testing, and again prior to treatment cohort entry. Safety and efficacy data were reviewed regularly by an Independent Data Monitoring Committee (IDMC). Trial oversight was provided by an independent Trial Steering Committee (TSC). This study is registered with ClinicalTrials.gov, NCT03182634; the European Clinical Trials database, EudraCT2015–003735–36; and the ISRCTN registry, ISRCTN16945804. The funders of the study (Cancer Research UK, AstraZeneca, Guardant Health, and Bio-Rad) had no role in study design, data collection, data analysis, data interpretation, or writing of the report. AstraZeneca reviewed the final version of the report but had no role in the decision to submit the manuscript for publication. The corresponding author had full access to all of the data and the final responsibility to submit for publication.

### Data availability statement

De-identified individual participant data, together with a data dictionary defining each field in the set, will be made available to other researchers on request, subject to the approval of a formal data access request in accordance with the ICR-CTSU data and sample access policy. Trial documentation including the protocol are available on request by contacting plasmamatch-icrctsu@icr.ac.uk.

The ICR-CTSU supports the wider dissemination of information from the research it conducts, and increased cooperation between investigators. Trial data is collected, managed, stored, shared, and archived according to ICR-CTSU Standard Operating Procedures to ensure the enduring quality, integrity, and utility of the data. Formal requests for data sharing are considered in line with ICR-CTSU procedures, with due regard given to funder and sponsor guidelines. Requests are via a standard proforma describing the nature of the proposed research and extent of data requirements.

Data recipients are required to enter a formal data sharing agreement, which describes the conditions for release and requirements for data transfer, storage, archiving, publication, and intellectual property. Requests are reviewed by the Trial Management Group (TMG) in terms of scientific merit and ethical considerations including patient consent. Data sharing is undertaken if proposed projects have a sound scientific or patient benefit rationale, as agreed by the TMG and approved by the TSC, as required.

Restrictions relating to patient confidentiality and consent will be limited by aggregating and anonymizing identifiable patient data. In addition, all indirect identifiers that may lead to deductive disclosures will be removed in line with Cancer Research UK Data Sharing Guidelines. Additional documents may be shared if approved by the TMG and TSC, e.g., statistical analysis plan and informed consent form.

## Results

### Baseline demographics and clinical characteristics

Between October 4, 2018 and August 27, 2020, 75 patients from 16 UK hospitals were recruited to Cohort E. 60/75 (80%) of patients had no mutation identified in ctDNA testing and 15/75 (20%) patients had ctDNA mutations (9 *PIK3CA*, 2 *PTEN*, 1 *ESR1*, 1 *HER2*, 1 *AKT1*, and 1 *PTEN/PIK3CA*) but there was no available cohort in plasmaMATCH at the time of treatment (Supplementary Fig. S1). The median age was 55.6 years [interquartile range (IQR), 45.7–64.1]; 42 (56%) patients had 1, and 13 (17%) had 2 prior line(s) of chemotherapy for metastatic disease, and 20 (27%) had no prior chemotherapy for advanced cancer having relapsed within 12 months of (neo)adjuvant chemotherapy. *BRCA1* and *BRCA2* germline mutations were present in 7 (9.3%) and 3 (4%) patients respectively. Three patients had somatic *BRCA*2 mutations (in the absence of germline *BRCA* mutations; Table 1). Further details regarding study population and geographical location are provided (Supplementary Tables S1–S3).

**Table 1.** Patients and characteristics.

	*N* = 75	
	*n*	%
Age, median:	55.6 years	
Metastatic at diagnosis		
Yes	12	16
No	63	84
Phenotype of primary tumor^a^		
HR negative HER2-negative	57	76
HR-positive HER2-negative	13	17.3
HR-positive HER2-positive	2	2.7
HR-negative HER2-positive	1	1.3
Systemic therapy for primary tumor		
Chemotherapy	56	88.9
Endocrine therapy	13	20.6
Anti-HER2 therapy	2	3.2
Platinum based chemotherapy	15	20
Phenotype of recurrence (or primary if recurrence not known)		
HR-negative HER2-negative	75	100
Disease sites		
Visceral	55	73.3
Soft tissue/nodal	20	26.7
Bone only	0	0
Systemic therapy for metastatic disease		
No chemotherapy	20	26.7
1 line chemotherapy	42	56
2 lines chemotherapy	13	17.3
Platinum-based chemotherapy	14	18.6
Immunotherapy	10	13.3
Endocrine therapy^b^	5	6.7
Anti-HER2 therapy^b^	1	6.7
Germline *BRCA* status		
No mutation	65	86.7
Pathogenic *BRCA1*	7	9.3
Pathogenic *BRCA2*	3	4
Somatic *BRCA* mutation^c^		
No mutation	70	93.3
Pathogenic *BRCA1*	0	0
Pathogenic *BRCA2*	3	4
Analyses in evaluable patients with no germline or somatic *BRCA* mutation	**55**	**73.3**
ATM loss		
No ATM loss	29	52.7
ATM loss	14	25.4
Inadequate or missing sample	12	21.8
Cyclin E1		
Cyclin E1 high	17	31
Cyclin E1 low	17	31
Inadequate or missing sample	21	38.1
RAD51		
RAD51 high	39	71
RAD51 low	3	5.4
Inadequate or missing sample	13	23.6
HRR gene status		
Wild-type	48	87.2
Germline mutation	2	3.6
Somatic mutation	4	7.3
Inadequate or missing sample	1	1.3

^a^Data not available for 2 patients; and where de novo stage IV disease: phenotype at presentation presented.

^b^The biopsy confirming triple-negative status was performed after this therapy.

^c^2 patients, no sample for analysis.

Further details regarding study population and geographical location are provided in Supplementary Tables S1–S3.

**Figure 1.**
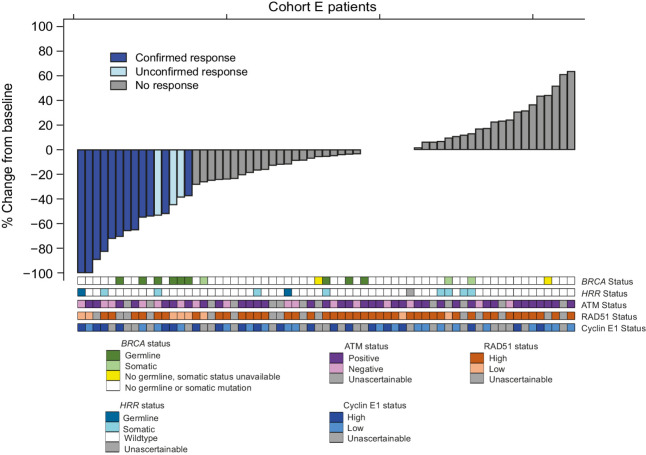
Best percentage change from baseline for sum of the target lesions (*n* = 70).

**Figure 2.**
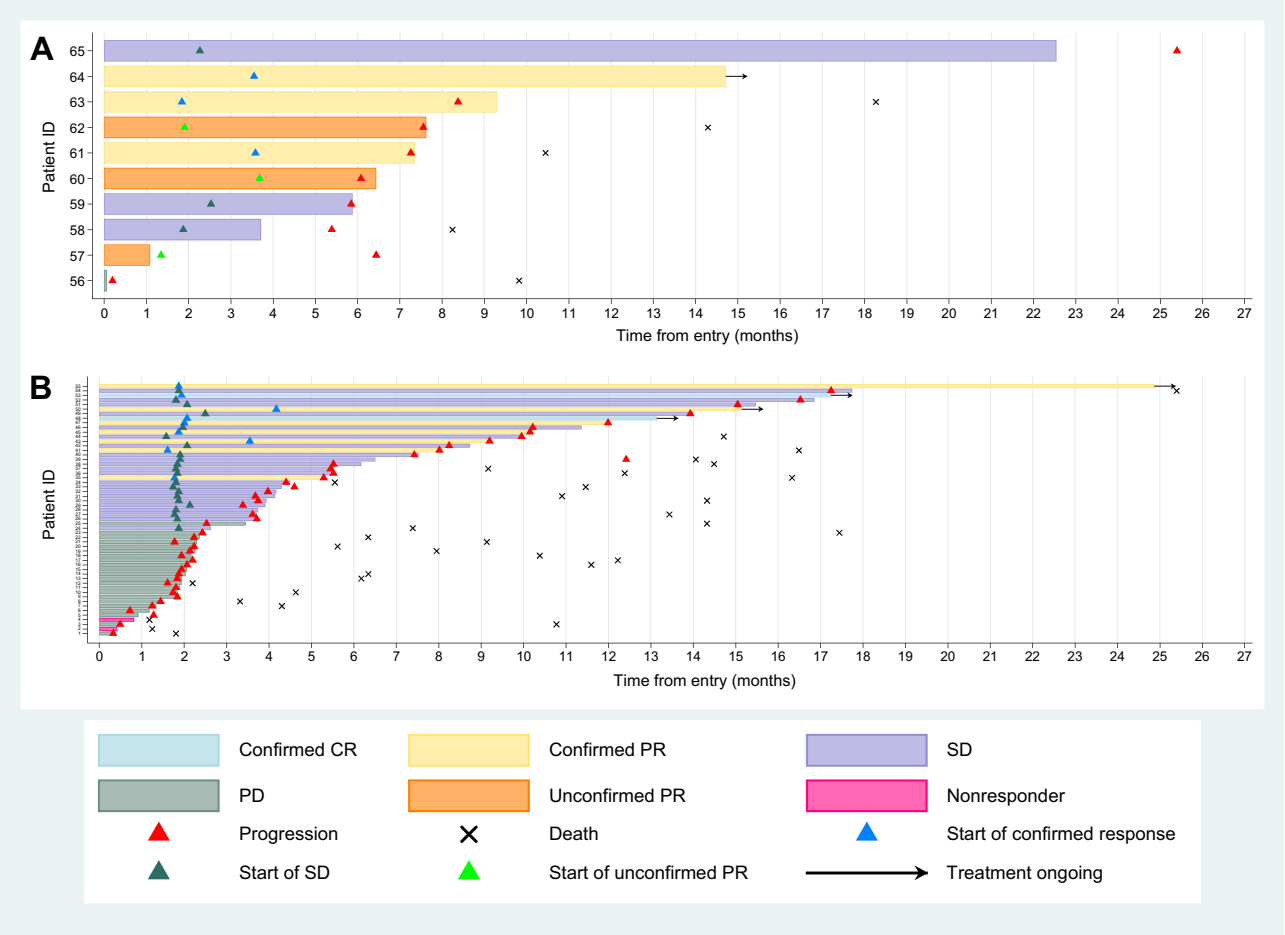
Swimmer plots for evaluable population with (**A**) germline *BRCA* mutations (*n* = 10) and (**B**) no germline or somatic *BRCA* mutations (*n* = 55).

Of the 75 patients entering Cohort E, 70 were evaluable for response; 3 patients never started treatment, 1 had no RECIST measurable disease at baseline and 1 patient did not have any post baseline RECIST scan data (Supplementary Fig. S1). The ORR (excluding unconfirmed responses) was 12/70; 17.1% (UMVUE = 18.1%; 95% CI, 10.4%–25.5%). All confirmed responses were in the first 69 evaluable patients. Median duration of response for the 12 patients who had confirmed CR/PR was 9.1 months IQR (6.0–11.5). Five patients with confirmed response were still on treatment at the time of the snapshot (Figs. 1 and 2).

In a *post hoc* analysis 16 of the 70 patients were confirmed as non-TNBC at diagnosis: the response rate in this group was 1/16; 6.3% (95% CI, 0.2–30.2) compared with 10/52; 19.2% (10/52; 95% CI, 9.6–32.5) for TNBC at diagnosis (Fisher exact *P* value = 0.44). There was no evidence of a significant difference in response rate between those patients with and without prior platinum exposure.

The clinical benefit rate was 21/70: 30% (exact 95% CI, 19.6–42.1). At a median follow-up of 18.3 months (IQR, 13.9–23.4), 59/75 (79%) patients had a PFS event with median PFS 4.3 months (IQR, 1.9–10.0 months; Supplementary Fig. S2) and 45 (60%) patients had died. To examine long-responders, an additional snapshot was taken on June 22, 2023, when 3 patients remained on treatment, with 48.6 (*BRCA* wild-type, no ATM loss, RAD51 high), 40.2 (germline *BRCA* mutation, ATM loss and RAD51 low) and 37.9 (*BRCA* wild-type, ATM loss, RAD51 unknown) months follow-up respectively.

### Biomarker analyses

**Figure 3.**
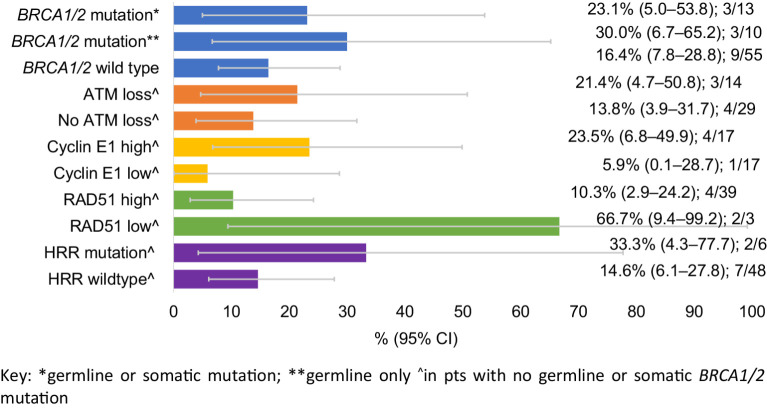
Activity according to biomarker subgroups: confirmed response rate.

In predefined subgroup analyses, confirmed response rate in patients with a *BRCA1/2* germline mutation was 3/10: 30% (exact 95% CI, 6.7%–65.2%), in patients with *BRCA1/2* germline or somatic mutations was 3/13: 23.1% (exact 95% CI, 5.0%–53.8%), and in patients with no *BRCA1/2* mutations (germline or somatic) was 9/55: 16.4% (exact 95% CI, 7.8%–28.8%; Fig. 3).

A subset of tumor tissues from 59 patients, were sequenced with a tissue panel, and no additional somatic *BRCA1/2* mutations were identified, supporting the primary somatic *BRCA1/2* analysis with Guardant360 on ctDNA.

As expected, low RAD51 was seen more frequently in patients with germline or somatic *BRCA* mutations (5/11; 45%) compared with those without *BRCA* mutations (3/42; 7%; Supplementary Table S4). RAD51 identified the two patients who showed a CR despite not harboring a *BRCA* mutation (Fig. 1): although one of these patients had a germline *PALB2* mutation. In patients without *BRCA1/2* mutations: ATM loss, high cyclin E1 expression, and functional HRD by RAD51 foci were associated with numerically higher rates of response, and PFS (Fig. 3; Table 2; and Supplementary Fig. S3).

**Table 2.** Activity according to biomarker subgroups: PFS (median, IQR, months).

	*N*	Median PFS (IQR), months
*BRCA1/2* mutation^a^	13	7.3 (4.5–25.4)
*BRCA1/2* mutation^b^	10	8.4 (6.1–25.4)
*BRCA1/2* wild-type	60	3.7 (1.9–10.0)
ATM loss^c^	14	3.4 (1.4–10.2)
No ATM loss^c^	33	2.5 (1.9–10.0)
Cyclin E1 high^c^	18	5.5 (2.5–10.2)
Cyclin E1 low^c^	18	2.2 (1.8–7.4)
RAD51 high^c^	42	2.5 (1.9–9.2)
RAD51 low^c^	4	Undetermined, median not reached

^a^Germline or somatic mutation.

^b^Germline only.

^c^In patients with no germline or somatic *BRCA1/2* mutation. Further data on activity in patients with BRCA mutations is provided in Supplementary Table S4.

Pathogenic mutations in HRR genes (not including germline and somatic *BRCA* mutations) were found in 10/74 (13.5%) patients (unascertainable in one patient; No patients had truncating *ATM* mutations in ctDNA analysis). In the 54 of these patients with evaluable disease and no germline or somatic *BRCA* mutations, 6 patients were found to have pathogenic mutations in *HRR* genes. The confirmed response rate in these patients HRR mutant cancer was 33% (2/6). A patient with a germline *PALB2* mutation had a CR, and a patient with somatic *NBN* R466fs mutation (also known as *NBS1*) a PR. In patients who were *BRCA* and *HRR* wild-type the response rate was 14.6% (7/48).

### Safety

**Figure 4.**
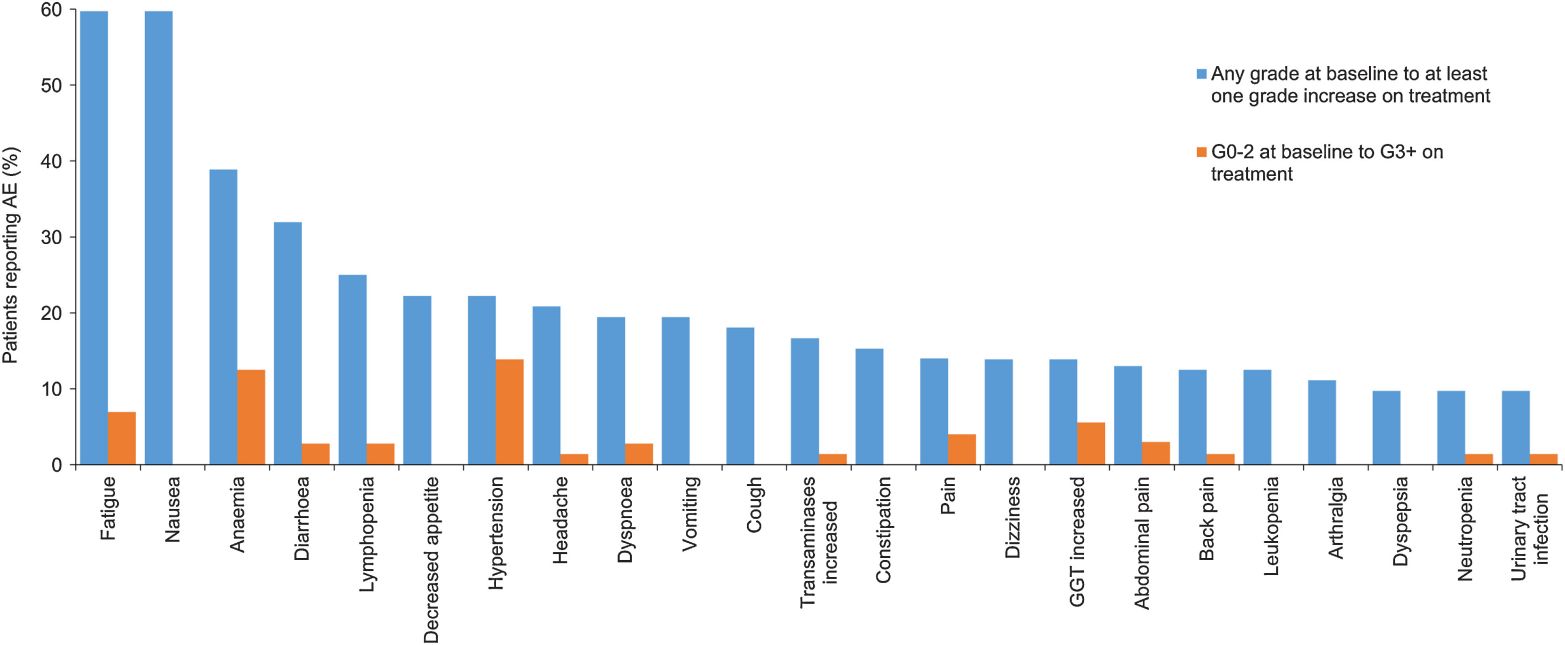
Treatment-emergent AEs more than 10% incidence.

Seventy-two patients were evaluable for the safety analysis (Fig. 4; Supplementary Table S5). Sixty-nine patients reported ≥ 1 treatment-emergent AEs at any grade. Thirty-two patients reported ≥ 1 treatment-emergent grade 3 or above AEs. The most common clinically significant treatment-emergent grade 3 AEs were hypertension (14%) and anemia (13%; Fig. 4).

Nineteen patients had a dose reduction in olaparib, and 14 patients had a dose reduction in ceralasertib. Six patients permanently discontinued treatment due to adverse effects; owing to diarrhoea, anemia requiring transfusion, chest pain, COVID-19, and fatigue (2 patients).

## Discussion

We report Cohort E of the plasmaMATCH study, a single group phase II study in patients with TNBC. The response rate to olaparib and ceralasertib did not meet prespecified criteria for efficacy in the overall evaluable population. However, additional biomarker work identified a number of potential predictive biomarkers, which could be investigated in future clinical trials.

Patients with germline and also somatic *BRCA1/2* mutations are known to respond to PARP inhibitors, such as olaparib, therefore the principal focus of the biomarker analysis was in patients with wild-type *BRCA1/2*. Patients had RAD51 foci assessed on the most recent biopsy, and although numbers are small, those patients without *BRCA1/2* mutations but with functional HRD by RAD51 foci responded to therapy (Fig. 3; One of these patients had a germline *PALB2* mutation). Tumors with high cyclin E1 protein may have evidence of replication stress, that could be exploited by olaparib plus ceralasertib therapy, and tumors with high cyclin E1 protein were more likely to benefit from therapy (Fig. 3). Although numerically higher response rates were seen in cancers with low ATM protein, the benefit seen in these patients were possibly lower than that observed with other ATR inhibitors or in preclinical models given in a more continuous schedule (18). Finally, in an exploratory analysis that was not pre-stated in the trial protocol, patients with other mutations in other HRR genes were potentially likely to benefit, with a CR in a patient with a germline *PALB2* mutation, further re-enforcing the role of PARP inhibition in patients with germline *PALB2* mutations (19).

Our study has a number of limitations. As a non-comparative phase II study we are unable to assess whether activity observed is a result of the combination therapy, or one of the individual drugs in the combination. In the recently presented VIOLETTE study (NCT03330847) patients with metastatic TNBC were randomized to receive olaparib, olaparib, and ceralasertib or olaparib and adavosertib (20). Patients were stratified on the basis of an HRR assay. In patients with no mutations (including no *BRCA* mutations) in the 15 HRR genes tested, the ORR to olaparib was 3.9% (2/51) and to olaparib and ceralasertib 15.4% (8/52), further indicating that responses to the combination are observed outside of the populations where existing biomarkers would predict activity. In the absence of a ceralasertib alone arm, the activity of ATR monotherapy is unknown.

Entry into Cohort E of plasmaMATCH was for patients who did not have actionable mutations in their ctDNA, or if no cohort was available, and this may have led to bias in patient recruitment. However, the cohort commenced late in the trial, the majority of patients were enrolled once other cohorts had stopped recruitment, and the patient population enrolled in Cohort E was likely overall reflective of unselected metastatic TNBC. Prior research has suggested that ATR inhibitors may have substantial efficacy in ATM-deficient cancers, yet in Cohort E we do not see strong evidence for activity in cancers with absent/low ATM expression. This may be reflective of selection on the basis of protein ATM expression, instead of selecting on the basis of ATM inactivating mutations, and/or may reflect the schedule of ATR inhibitor employed, with potentially more continuous schedules being more optimal for ATM-deficient cancers.

In conclusion, we report here the result of a large phase II study in advanced TNBC with olaparib plus ceralasertib therapy. The study did not observe sufficient evidence of efficacy by the predefined criteria, and this is broadly in line with the data observed in the randomized phase II VIOLETTE study (20). Nevertheless, we identify a number of biomarkers of potential benefit, in particular functional HRD by RAD51 foci, high cyclin E1 expression and pathogenic mutations in HRR genes. Further research is warranted to investigate the potential benefit of olaparib, or olaparib plus ceralasertib therapy, in these biomarker defined subset of patients with advanced TNBC.

## Supplementary Material

Supplementary Figure S1 Figure S1. CONSORT diagram

Supplementary Figure S2 Figure S2. Progression free survival Kaplan Meier curve

Supplementary Figure S3 Figure S3. Best percentage change from baseline for sum of the target lesions (n=70) by BRCA mutation status

Supplementary Table S1 Table S1. Additional baseline characteristics

Supplementary Table S2 Table S2.Representativeness of Study Participants

Supplementary Table S3 Table S3. List of centres and recruitment

Supplementary Table S4 Table S4: Activity according to biomarker subgroups: Progression free survival (median, IQR, months) in patients with BRCA1/2 mutations (germline or somatic)

Supplementary Table S5 Table S5. Worst grade CTCAE reported during treatment
