# Supplementary material for: Olaparib and Ceralasertib (AZD6738) in Patients with Triple-Negative Advanced Breast Cancer: Results from Cohort E of the plasmaMATCH Trial (CRUK/15/010)
Source: Clin Cancer Res. 2023 Sep 29;29(23):4751–9. doi: 10.1158/1078-0432.CCR-23-1696 (PMC10690092; doi:10.1158/1078-0432.CCR-23-1696)

Olaparib and Celarasertib (AZD6738) in patients with triple negative advanced breast cancer: results from Cohort E of the plasmaMATCH trial (CRUK/15/010)

Supplementary material

Figure S1. CONSORT diagram

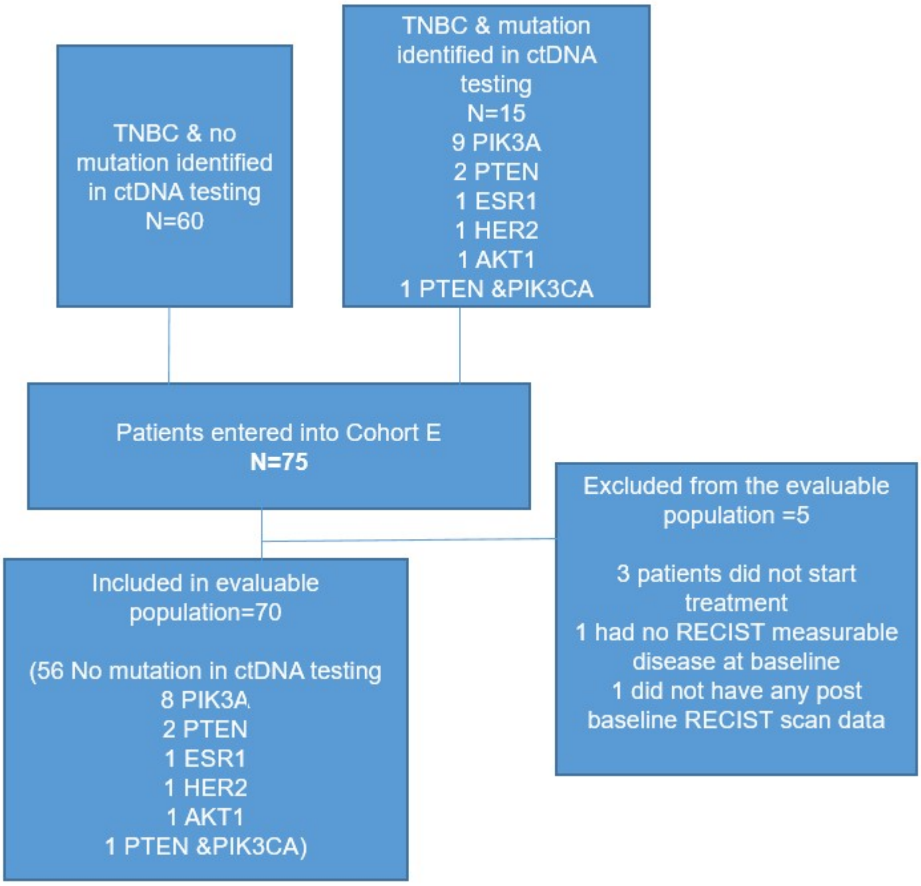

Supplement: Supplementary Figure S1 — Figure S1. CONSORT diagram [file ccr-23-1696_supplementary_figure_s1_suppfs1.pdf]
