# Supplementary material for: Olaparib and Ceralasertib (AZD6738) in Patients with Triple-Negative Advanced Breast Cancer: Results from Cohort E of the plasmaMATCH Trial (CRUK/15/010)
Source: Clin Cancer Res. 2023 Sep 29;29(23):4751–9. doi: 10.1158/1078-0432.CCR-23-1696 (PMC10690092; doi:10.1158/1078-0432.CCR-23-1696)

Figure S3. Best percentage change from baseline for sum of the target lesions (n=70) by BRCA mutation status

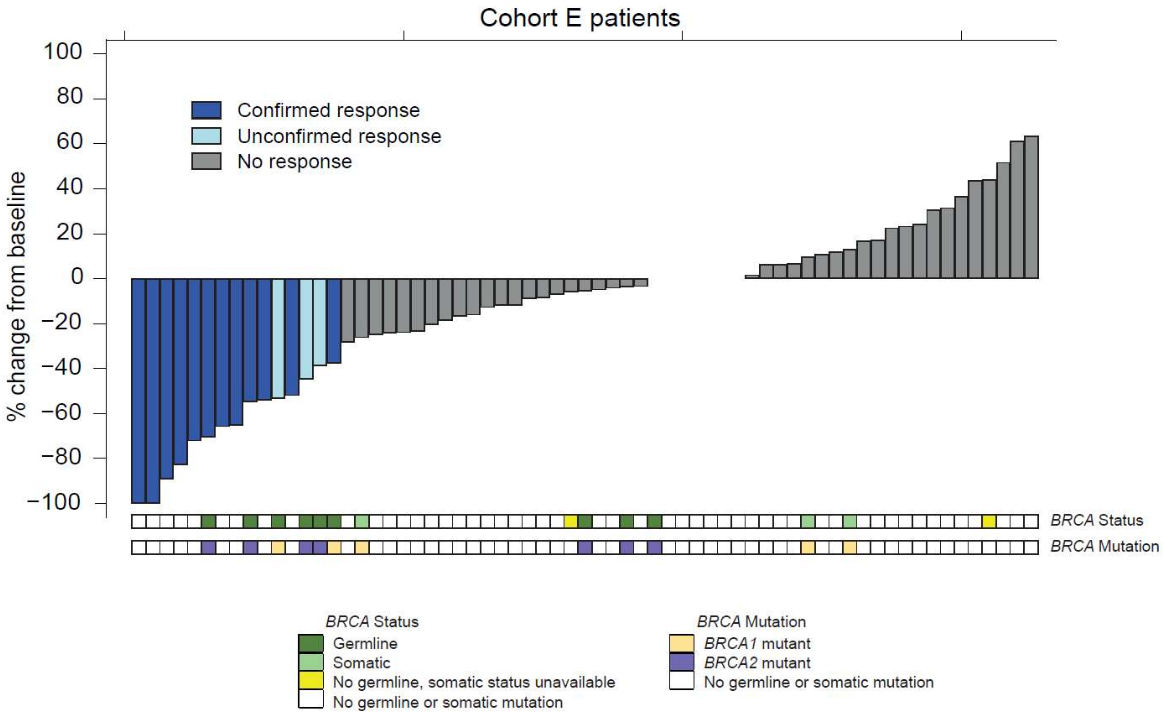

Supplement: Supplementary Figure S3 — Figure S3. Best percentage change from baseline for sum of the target lesions (n=70) by BRCA mutation status [file ccr-23-1696_supplementary_figure_s3_suppfs3.pdf]
