# Supplementary material for: Olaparib and Ceralasertib (AZD6738) in Patients with Triple-Negative Advanced Breast Cancer: Results from Cohort E of the plasmaMATCH Trial (CRUK/15/010)
Source: Clin Cancer Res. 2023 Sep 29;29(23):4751–9. doi: 10.1158/1078-0432.CCR-23-1696 (PMC10690092; doi:10.1158/1078-0432.CCR-23-1696)
Supplement: Supplementary Table S1 — Table S1. Additional baseline characteristics [file ccr-23-1696_supplementary_table_s1_suppts1.pdf]

Table S1. Additional baseline characteristics

	N=75	
	**n**	**%**
**Sex**		
Female	75	100
**Age group (years) at registration**		
<50	26	34.7
≥50 & <60	21	28
≥60 & <70	20	26.7
≥70	8	10.7
**Ethnicity**		
White: British	67	89.3
White: Any other White background	3	4
Asian or Asian British: Indian	1	1.3
Black or Black British: Caribbean	2	2.7
Black or Black British: Any other Black background	1	1.3
Chinese or other ethnic group: Chinese	1	1.3
