# Supplementary material for: Olaparib and Ceralasertib (AZD6738) in Patients with Triple-Negative Advanced Breast Cancer: Results from Cohort E of the plasmaMATCH Trial (CRUK/15/010)
Source: Clin Cancer Res. 2023 Sep 29;29(23):4751–9. doi: 10.1158/1078-0432.CCR-23-1696 (PMC10690092; doi:10.1158/1078-0432.CCR-23-1696)
Supplement: Supplementary Table S2 — Table S2.Representativeness of Study Participants [file ccr-23-1696_supplementary_table_s2_suppts2.pdf]

Table S2.Representativeness of Study Participants

**Cancer type/subtype/stage**	**Breast cancer/Triple negative/Advanced**
**Considerations related to:**	
Sex	TNBC, as all other subtypes of breast cancer, is a predominantly female disease and is rare in men. Male breast cancer represents only between 0.5 and 1% of all breast cancers diagnosed each year. TNBC incidence is around 10-15% among all breast cancer subtypes in the overall largely female population (Breast Cancer Res and Treatment 2008;1132(2):357-60).
Age	The median age of presentation of breast cancer in the UK is 62 years in white patients and 50 years in black patients (www.ncin.org.uk). Patients with TNBC present at a younger age, than the overall population of patients with breast cancer. (Cancer 2019;125(19):3412-3417)
Race/Ethnicity	In England, 87% of breast cancer diagnoses are made in women who are white, and 7% in non-white women (6% unknown). (British Journal of Cancer 2022;1765-1773). Incidence rates for breast cancer are lower in the Asian and Black ethnic groups, and in people of mixed or multiple ethnicity compared with the white ethnic group. However, TNBC is known to be more common in non-white women (Cancer 2019;125(19):3412-3417).
Geography	The incidence rate of breast cancer varies in different geographical regions on England (between 162 and 183 per 100,000 people, from lowest to highest). Other social determinants (such as poverty, lack of education, social isolation) also influence breast cancer incidence, treatment and survival.
**Overall representativeness of the study:**	
The median age of patients in this study was 57 years, similar to the average age distribution in the literature. Eleven percent of patients in this study were non-white, representing the population distribution of breast cancer, although this may be a slight under-representation of the population of women with TNBC. Patients were recruited from 20 sites: representing a broad range of urban and rural and social demographics across England, Scotland and Wales.	
