# Supplementary material for: Olaparib and Ceralasertib (AZD6738) in Patients with Triple-Negative Advanced Breast Cancer: Results from Cohort E of the plasmaMATCH Trial (CRUK/15/010)
Source: Clin Cancer Res. 2023 Sep 29;29(23):4751–9. doi: 10.1158/1078-0432.CCR-23-1696 (PMC10690092; doi:10.1158/1078-0432.CCR-23-1696)
Supplement: Supplementary Table S3 — Table S3. List of centres and recruitment [file ccr-23-1696_supplementary_table_s3_suppts3.pdf]

Table S3. List of centres and recruitment

Centre Name	Total
Royal Marsden Hospital, London	8
Royal Marsden Hospital, Sutton	7
Royal Bournemouth Hospital, Bournemouth	4
University College London Hospital, London	3
Addenbrooke's Hospital, Cambridge	2
West of Scotland Beatson Cancer Centre, Glasgow	9
Royal Devon and Exeter, Exeter	4
The Christie Hospital, Manchester	15
Western General Hospital, Edinburgh	1
Barts Health NHS Trust, London	0
Oxford University Hospitals NHS Trust, Oxford	1
Velindre Cancer Centre, Cardiff	1
Royal Cornwall Hospital, Truro	0
Weston Park Hospital, Sheffield	6
University Hospitals Bristol NHS Foundation Trust, Bristol	2
University Hospital Southampton, Southampton	6
Derriford Hospital, Plymouth	1
Nottingham University Hospitals NHS Trust, Nottingham	0
Clatterbridge Cancer Centre, Clatterbridge	1
Kent Oncology Centre, Maidstone	4
	75
