# Supplementary material for: Olaparib and Ceralasertib (AZD6738) in Patients with Triple-Negative Advanced Breast Cancer: Results from Cohort E of the plasmaMATCH Trial (CRUK/15/010)
Source: Clin Cancer Res. 2023 Sep 29;29(23):4751–9. doi: 10.1158/1078-0432.CCR-23-1696 (PMC10690092; doi:10.1158/1078-0432.CCR-23-1696)
Supplement: Supplementary Table S4 — Table S4: Activity according to biomarker subgroups: Progression free survival (median, IQR, months) in patients with BRCA1/2 mutations (germline or somatic) [file ccr-23-1696_supplementary_table_s4_suppts4.pdf]

Table S4: Activity according to biomarker subgroups: Progression free survival (median, IQR, months) in patients with *BRCA1/2* mutations (germline or somatic)

	Confirmed response rate % (95%CI); n/N	N	Median PFS (IQR), months
**ATM loss**	33.3 (0.8, 90.6); 1/3	3	6.1 (6.1, 8.4)
**No ATM loss**	22.2 (2.8, 60.0); 2/9	9	7.3 (4.5, 25.4)
**Cyclin E1 high**	25.0 (0.6, 80.6); 1/4	4	7.3 (4.3, 25.4)
**Cyclin E1 low**	33.0 (0.8, 90.6); 1/3	3	6.1 (6.1, 8.4)
**RAD51 high**	16.7 (0.4, 64.1); 1/6	6	8.4 (5.8-25.4)
**RAD51 low**	20.0 (0.5, 71.6); 1/5	5	6.1 (4.5-undetermined)
