# Supplementary material for: Olaparib and Ceralasertib (AZD6738) in Patients with Triple-Negative Advanced Breast Cancer: Results from Cohort E of the plasmaMATCH Trial (CRUK/15/010)
Source: Clin Cancer Res. 2023 Sep 29;29(23):4751–9. doi: 10.1158/1078-0432.CCR-23-1696 (PMC10690092; doi:10.1158/1078-0432.CCR-23-1696)
Supplement: Supplementary Table S5 — Table S5. Worst grade CTCAE reported during treatment [file ccr-23-1696_supplementary_table_s5_suppts5.pdf]

Table S5. Worst grade CTCAE reported during treatment

Note: AEs reported in the table below are where the AE was reported for ≥10% patients at any grade or where at least one patient has a grade 3+ AE. For a full list of AEs see appendix.

	G0		G1		G2		G3		G4		G5		Any grade		G3+	
	n	%	n	%	n	%	n	%	n	%	n	%	n	%	n	%
Abdominal pain	63	88	5	7	1	1	3	4	0	0	0	0	9	13	3	4
Anaemia	44	61	10	14	9	13	9	13	0	0	0	0	28	39	9	13
Arthralgia	62	86	8	11	2	3	0	0	0	0	0	0	10	14	0	0
Back pain	60	83	5	7	6	8	1	1	0	0	0	0	12	17	1	1
Chest pain	66	92	4	6	1	1	1	1	0	0	0	0	6	8	1	1
Constipation	60	83	10	14	2	3	0	0	0	0	0	0	12	17	0	0
Corona virus infection	71	99	0	0	0	0	0	0	0	0	1	1	1	1	1	1
Cough	56	78	12	17	4	6	0	0	0	0	0	0	16	22	0	0
Decreased appetite	55	76	13	18	4	6	0	0	0	0	0	0	17	24	0	0
Diarrhoea	49	68	19	26	2	3	1	1	1	1	0	0	23	32	2	3
Dizziness	62	86	6	8	4	6	0	0	0	0	0	0	10	14	0	0
Dyspepsia	63	88	5	7	4	6	0	0	0	0	0	0	9	13	0	0
Dyspnoea	56	78	8	11	6	8	1	1	1	1	0	0	16	22	2	3
Fatigue	21	29	31	43	15	21	5	7	0	0	0	0	51	71	5	7
Febrile neutropenia	71	99	0	0	0	0	1	1	0	0	0	0	1	1	1	1
Gamma-glutamyltransferase increased	62	86	4	6	2	3	4	6	0	0	0	0	10	14	4	6
Haemoglobin decreased	71	99	0	0	0	0	1	1	0	0	0	0	1	1	1	1
Headache	57	79	13	18	1	1	1	1	0	0	0	0	15	21	1	1
Hot flush	65	90	5	7	2	3	0	0	0	0	0	0	7	10	0	0
Hypertension	51	71	4	6	5	7	12	17	0	0	0	0	21	29	12	17
Hypokalaemia	71	99	0	0	0	0	0	0	1	1	0	0	1	1	1	1
Hyponatraemia	71	99	0	0	0	0	1	1	0	0	0	0	1	1	1	1
Intestinal perforation	71	99	0	0	0	0	1	1	0	0	0	0	1	1	1	1

	G0		G1		G2		G3		G4		G5		Any grade		G3+	
	n	%	n	%	n	%	n	%	n	%	n	%	n	%	n	%
Leukopenia	63	88	6	8	3	4	0	0	0	0	0	0	9	13	0	0
Lymphopenia	53	74	8	11	9	13	2	3	0	0	0	0	19	26	2	3
Mucosal inflammation	71	99	0	0	0	0	1	1	0	0	0	0	1	1	1	1
Nausea	29	40	32	44	11	15	0	0	0	0	0	0	43	60	0	0
Neutropenia	65	90	5	7	1	1	0	0	1	1	0	0	7	10	1	1
Neutropenic sepsis	71	99	0	0	0	0	1	1	0	0	0	0	1	1	1	1
Oedema peripheral	68	94	2	3	1	1	1	1	0	0	0	0	4	6	1	1
Pain	61	85	4	6	4	6	3	4	0	0	0	0	11	15	3	4
Pulmonary embolism	71	99	0	0	0	0	1	1	0	0	0	0	1	1	1	1
Thrombocytopenia	66	92	4	6	1	1	1	1	0	0	0	0	6	8	1	1
Transaminases increased	59	82	10	14	2	3	1	1	0	0	0	0	13	18	1	1
Urinary tract infection	65	90	2	3	4	6	1	1	0	0	0	0	7	10	1	1
Vomiting	58	81	11	15	3	4	0	0	0	0	0	0	14	19	0	0
